# Supplementary material for: Identification of photoperiod-induced specific miRNAs in the adrenal glands of Sunite sheep (Ovis aries)
Source: Front Vet Sci. 2022 Jul 22;9:888207. doi: 10.3389/fvets.2022.888207 (PMC9354845; doi:10.3389/fvets.2022.888207)
Supplement: Supplementary file 1 [file Data_Sheet_1.ZIP › Supplementary file/miRDeep2 file.zip!/miRDeep2 file/Know_Structure/LP42A1/oar-let-7i.pdf]

miRBase precursor : oar-let-7i
Total read count : 435996
oar-let-7i read count : 435785
remaining reads : 211

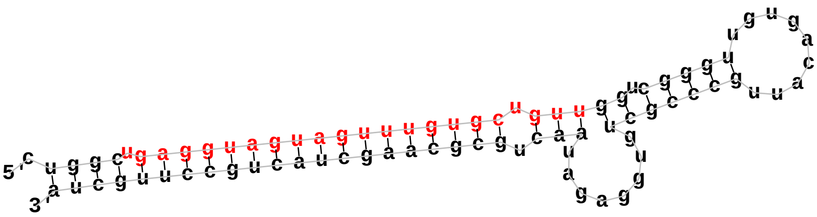

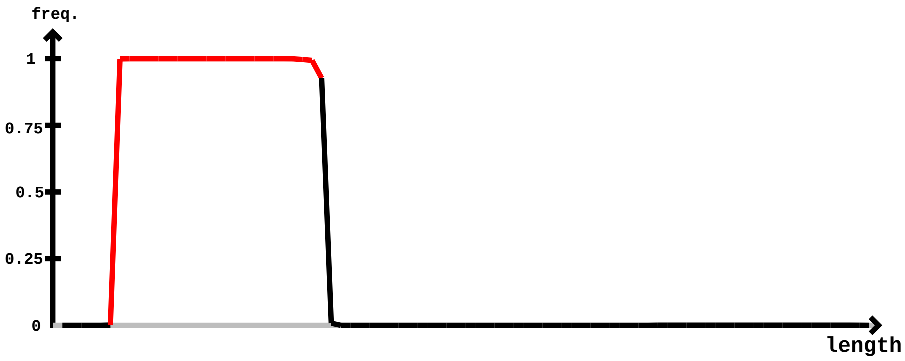


```
       oar-let-7i
5'- cuggcugagguaguaguuugugcuguuggucggguugugacauugcccgcuguggagauaacugcgcaagcuacugccuugcua   -3'    exp
    .((((.(((((((((((((((((.(((((.(((((.........)))))))........))).)))))))))))))))))))))    reads      mm         sample
    ....cugagguaguaguuugu...............................................................    1          0          seq
    ....cugagguaguaguuugugcug...........................................................    2          0          seq
    ....cugagguaguaguuugugcugu..........................................................    28         0          seq
    ....cugagguaguaguuugugcuguu.........................................................    396        0          seq
    ....cugagguaguaguuugugcuguug........................................................    1          0          seq
    .....ugagguaguaguuugug..............................................................    6          0          seq
    .....ugagguaguaguuugugA.............................................................    11         1          seq
    .....ugagguaguaguuugugc.............................................................    69         0          seq
    .....ugagguaguaguuugugG.............................................................    1          1          seq
    .....ugagguaguaguuugugcu............................................................    1075       0          seq
    .....ugagguaguaguuGgugcu............................................................    1          1          seq
    .....ugagguaguaguuugugcug...........................................................    1463       0          seq
    .....ugagguaguaguuugugcugu..........................................................    28982      0          seq
    .....ugagguaguaguuugugcuguu.........................................................    400071     0          seq
    .....ugagguaguaguuugugcuguug........................................................    3436       0          seq
    .....ugagguaguaguuugugcuguugg.......................................................    19         0          seq
    .....ugagguaguaguuugugcuguuggu......................................................    2          0          seq
    ......gagguaguaguuugugc.............................................................    1          0          seq
    ......gagguaguaguuugugcugu..........................................................    15         0          seq
    ......gagguaguaguuugugcuguu.........................................................    204        0          seq
    .........guaguaguuugugcuguuU........................................................    1          1          seq
    .............................................................cugcgcaagcuacugccuug...    7          0          seq
    .............................................................cugcgcaagcuacugccuugc..    80         0          seq
    .............................................................cugcgcaagcuacugccuugcu.    123        0          seq
    ..............................................................ugcgcaagcuacugccuugcu.    1          0          seq
```
